# Editorial: Social sustainability at work: A key to sustainable development in business

**DOI:** 10.3389/fpsyg.2022.1108935

**Published:** 2022-12-23

**Authors:** Susanne Rank, Francoise Contreras, Ghulam Abid

**Affiliations:** ^1^School of Business, Mainz University of Applied Sciences, Mainz, Germany; ^2^School of Management and Business, Universidad del Rosario, Bogota, Colombia; ^3^Department of Business Studies, Kinnaird College for Women, Lahore, Pakistan

**Keywords:** social sustainability at work, social development in business, CSR, social performance, green HRM

## Introduction

The Sustainable Development Goals (SDG) were adopted by the UN in 2015 as part of the 2030 Agenda for Sustainable Development [United Nations (UN), 2022]: “These SDGs are a universal call to action to end poverty, protect the planet and improve the lives and prospects of everyone, everywhere”. The SDGs are first mentioned in the Brundtland Report in 1987 as sustainable development should “meet their needs without compromising the ability of future generations to meet their own needs”. Thirty-five years later, the climate change became irreversible with a dramatic impact on poverty, emigration from hot regions (e.g., Madagascar), and future social conflicts worldwide. Currently, Randers et al. (2018) provide four scenarios and the subsequent consequences for societies within given planetary boundaries and proactive measures by 2030 on limiting the global warming below 2°C. The rapid industrialization, global warming, the health crisis, and the political instability of the world, among other important recent events, show the urgent necessity of having sustainable companies (Okumus et al., 2019; Duric and Potocnik Topler, 2021). This explains the broad scope of sustainable development with SDGs and their call for urgency, especially in the field of business and management.

In the business context, social sustainability is key for sustainable development and serves as a measure of people's wellbeing and social engagement within the organization and the community, as the current climate transformation largely impacts social issues in society like poverty or migration to healthy climate zones. Social sustainability applied to organizations contributes to employees' wellbeing and health through the construction of sustainable places to work characterized by positive work environments as the major focus of SDGs, mainly those related to good health and wellbeing (SDG 3) as part of an integrated approach, decent work and economic growth (SDG 8), and gender equality (SDG 5, United Nations, 2015). However, these goals are closely interlinked with one another. They share as a concern and goal the call for help for a lasting planet with better quality of life standards for its inhabitants.

Within this frame of reference, business environment and organizational contexts become particularly relevant in making an important contribution to sustainability. Nowadays, companies have to operate in a complex environment with new contingencies and challenges to be competitive and to last. There is no doubt about the extent to which the pandemic changed the way people work and live (Rigotti et al., 2021). Currently, there is an accelerated digital transformation that has changed and is changing the way companies work and should be managed (Hanelt et al., 2021). However, it is important to acknowledge that the COVID-19 pandemic health crisis not only brought difficulties, it also taught us new ways to live and work. The crisis offers huge opportunities that can make companies more competitive if considered. For example, companies are currently more aware of the importance of creating inclusive work environments, resulting in a high innovation rate through a diverse workforce. Framed in social responsibility, organizations have the opportunity to contribute not only to mitigating the difficult conditions of forced migration the crisis has led to, but to also enhancing their innovation and creativity through inclusive work environments. Thus, innovation makes companies globally competitive in a highly complex and uncertain business environment while promoting a better society (Voegtlin et al., 2022).

In the work context, there is a consensus among researchers about the sparse considerations and commitments that have been presented toward social sustainability, but this approach has received less attention (Torkayesh et al., 2021), because the sustainable perspective has underestimated the human and social factors that such a perspective involved (Magis and Shinn, 2008; Vallance et al., 2011). Social sustainability includes people's health and safety, community engagement, philanthropic actions, corporate citizenship, corporate governance, the supply chain, and employee working conditions (Hedstrom, 2018). We as editors propose that social science can be crucial to supporting long-term sustainability in business and healthy lives at work, as the research articles of this Research Topic show.

In this context, organizations can contribute to achieving the SDGs through ethical behavior and corporate social responsibility (CSR, Carroll, 1979). CSR is based on the stakeholder approach to sustainable business by achieving an obligation to internal vs. external stakeholders, impacting our society and our environment (Carroll, 1979). In academic reviews (Kolk, 2016; Pisani et al., 2017; Turner et al., 2019), the impact of CSR pillars on business outcomes (employees, customers, and financial) is given. Beyond profit making, companies have to act collectively as responsible social actors that ethically engage with their internal vs. external stakeholders, comply with environmental and labor standards, and respect governance, politics, and customs. Although companies have to integrate social and environmental factors in their financial decisions, contributing to building a fairer economy with a local and global sustainable impact might be a radical mental shift and learning journey for CEOs (Business and Sustainable Development Commission, 2017). Thus, the key challenge is how to globally tackle and balance the economic success with environmental and social demands.

Furthermore, organizational and employee outcomes could be fostered by sustainable business strategies, policies, and practices aimed toward this end. The benefits of CSR framework result on the organizational level in higher organizational commitment, work engagement, increased social-communal, green, and economic performance, and organizational citizenship. If these social sustainability strategies are implemented by Human Resource Management (HRM), high employee performance, individual psychological empowerment, and wellbeing are developed on the individual level (Chams and García-Blandón, 2019; Turner et al., 2019; Wang et al., 2020; Paulet et al., 2021). Aust et al. (2020) pointed out that specific sustainable HRM practices affect organizational vs. individual behavior by an outside-in approach to humans in society and to the company, and finally support the SDG (United Nations, 2022).

## Studies overview for the Research Topic “social sustainability at work and in business”

In our Research Topic, business success and organizational behavior from the social sustainability approach by teams and individuals in organizations are addressed from different research perspectives in various countries. Our international colleagues enrich this Research Topic by contributing to different foci on sustainable development, especially on creativity, innovation, wellbeing, mental health, work engagement, and CSR. This Research Topic comprises 16 articles that address sustainable development in the business field from different approaches, which are briefly introduced.

From a wider perspective, in their research on “*The psychological concept of social sustainability in the workplace from the perspective of sustainable goals: A systematic review*” Kobal Grum and Babnik conducted a systematic literature review to understand the phenomenon of social sustainability at the workplace framed in SDGs. Based on the main topics identified from their analysis, they present a theoretical model describing the psychological concept of social sustainability at the workplace from the perspective of SDGs.

At the organizational level, in the paper “*Talent acquisition and technology: A step toward sustainable development*” Rehman, Ullah et al. offer a conceptual and empirical model of employee recruitment through the use of social media and information technology as a step toward sustainable development. Following this level of analysis, Baykal and Bayraktar conducted research on “*Effects of green human resources management practices on work engagement: Mediating effect of psychological ownership*”. The authors found that this green HRM approach can lead to higher employee work engagement. Likewise, they verified the mediator role of psychological ownership in such a relationship. In this same vein, in the article “*Mediating role of green supply chain management between lean manufacturing practices and sustainable performance*” Awan et al. analyzed green supply chain management. They demonstrate that process and equipment, product design, supplier relationships, and customer relationships have a significant effect on sustainable performance. Innovation was also studied from this same organizational approach. In the study “*Linkages between knowledge management process and corporate sustainable performance of Chinese SMEs: mediating the role of frugal innovation*” Kun analyzed the effect of the knowledge management process on sustainable corporate performance with the association of frugal innovation. The author found that all dimensions of knowledge management have a significant impact on corporate sustainable performance. In addition, frugal innovation has a significant impact on corporate sustainable performance.

As a crucial factor of business sustainability, CSR was addressed by Wang and Bian in the study of “*Analyzing the role of corporate social responsibility for sustainable environmental performance: Mediating roles of environmental strategy and environmental outcomes*”. Among other findings, CSR influences environmental performance and is positively correlated with environmental strategy and environmental outcomes, which in turn improve environmental performance. Likewise, in the paper “*Fostering advocacy behavior of employees: A CSR perspective from the hospitality sector*” Ahmad et al. provide evidence on how CSR perceptions of hotel employees can drive their advocacy behavior. As they proposed, hotels can improve their reputation by converting their employees into advocates by investing in hotels' CSR commitment to enhance employees' engagement in their advocacy behavior. Finally, also linked to CSR, in his research “*Responsible leadership and affective organizational commitment: The mediating effect of corporate social responsibility*” Piñeros Espinosa found that CSR mediated the influence of responsible leadership on affective organizational commitment. He proposes responsible leadership as a valid mechanism to develop CSR practices and finally increase the employees' affective organizational commitment.

Further studies focus on the negative employee experiences that can affect their wellbeing. In this regard, in his research paper on “*Impact of work demand constraints on psychological distress through workplace bullying: A moderated-mediation model*” Naseem demonstrated that work demand constraints play a significant role in workplace bullying. Thus, this bullying heightens the employees' psychological distress. Additionally, violence against women also was studied. In the study about “*Effects of intimate partner violence against women in international micro and small enterprises relationships: The mediator role of capabilities*” Ponce-Gómez et al. analyzed a group of women owners of exporting MSEs in Peru: Intimate Partner Violence Against Women (IPVAW) influences the export capabilities and the quality of the relationships that women maintain with importers and suppliers. Finally, in the study on “*Impact of COVID-19 and consortium factors on mental health: Role of emotional labor strategies in achieving sustainable development goals*” Rehman, Hamza et al. address the United Nation's SDGs related to decent work and economic growth. They highlighted the importance of maintaining employees' mental health and psychological stability.

Moreover, Santana-Martins et al. focused how leadership impacts employees' wellbeing and organizational outcomes in their article “*Employees' emotional awareness as an antecedent of organizational commitment—The mediating role of affective commitment to the leader*”. As affective commitment in the workplace is crucial to businesses' sustainability, they analyze employees' emotional awareness as an antecedent of commitment and probe the mediating role of affective commitment in this relationship. In the same vein, in the study on “*Linking authentic leadership to transactive memory system, team innovativeness and selling performance: A multilevel investigation*” Shahzad, Iqbal, Akbar et al. analyze the impact of authentic leadership on salespersons' behavior in a B2B selling context. The authors found that authentic leadership behavior has a stronger relationship with the transactive memory system, innovative work behavior, and customer-directed OCB. Furthermore, in their next study on “*The role of transformational leadership on firm performance: Mediating effect of corporate sustainability and moderating effect of knowledge-sharing*” Shahzad, Iqbal, Jan et al. demonstrated that knowledge-sharing has a moderating role in the relationship between transformational leadership and firm performance. They highlight the significant role of leadership style on firm performance and knowledge-sharing culture. In a further study on the “*Eminence of leader humility for follower creativity during COVID-19: The role of self-efficacy and proactive personality*” Asghar et al. highlight the importance of leader humility for improving creativity during the COVID-19 pandemic. Finally, the economic perspective was addressed by Farooq et al. in their study on the “*Surge in economic growth of Pakistan: A case study of china Pakistan economic corridor*”. They analyze the China-Pakistan Economic Corridor (CPEC) and its impact on the economic growth of Pakistan by analyzing macroeconomic variables. The key results show that foreign direct investment and human capital investment have a positive effect on the economic growth of Pakistan.

Finally, we highlighted the importance of social and environmental sustainability, as Kemp et al. (2022) say that “the common strength and benefit of investing in sustainable behavior in all organizations is the mitigation of the global high risk of failing to save our planet with respect to the lives of our future generations and children”. We hope the readers enjoy the different research perspectives while reading these articles and findings, and, furthermore, that these findings inspire the implementation of some sustainable actions in organizations now.

## Author contributions

All authors listed have made a substantial, direct, and intellectual contribution to the work and approved it for publication.
